# A Practical Treatise on the Diseases, Injuries, and Malformations of the Urinary Bladder, the Prostate Gland and the Urethra

**Published:** 1855-08

**Authors:** 


ART. V.—*A Practical Treatise on the Diseases, Injuries, and Malformations of the Urinary Bladder, the Prostate Gland and the Urethra.* By S. D. GROSS, M. D., Prof. of Surgery in the University of Louisville, one of the Surgeons of the Louisville Marine Hospital, etc., etc. Second edition, Revised and much enlarged. With one hundred and eighty-four illustrations. Philadelphia: Blanchard and Lea. 1855.

The addition of some two hundred pages, and many illustrations, to Prof. Gross's work, has transformed it into a large octavo of more than nine hundred pages, presenting, typographically, that creditable appearance which it is the habit of Messrs. Blanchard and Lea to bestow upon their publications.

We have often expressed our predilection for the monograph, as the proper method of concentrating and diffusing medical knowledge. And in no instance has this preference been more fully justified than in this admirable work. It fills a hiatus. No monographist, save Gross, has given anything like a full treatise on this specialty, and when we reflect that this work, though full, is not redundant, that it has no verbiage, no waste matter, while it exhausts the subject, we may at once appreciate its merit and value.

The five opening chapters are devoted to the surgical anatomy of the perineum, of the bladder, of the prostate gland, of the urethra, and to the nature and composition of the urine. It is enough to say of these that the descriptions are clear, accurate, concise, and well illustrated.

The body of the work begins with a chapter on malformations of the bladder. Many instances of absence of the bladder, of its bilobation, and of extrophy of the organ, are recorded, with such scanty suggestions for treatment, management, or surgical relief, as these almost hopeless cases can admit.

Upon wounds of the bladder our author is satisfactorily full. Heretofore very little information on this subject has existed in surgical works; the little that is to be found being confined to isolated cases in our periodical literature, or to surgical histories, like Larrey's Memoirs of Military Surgery, and the works of M. Percy and G. J. Guthrie, on Army Surgery. From these various sources, as well as from personal experience, Prof. Gross has collected a sufficient number of cases to show that these accidents are not so fatal as would seem likely at first glance. Where the wound is small or tortuous in its course, the fatality is greater, owing to the greater liability of urinary infiltration. Again, wounds above the pubis, being more likely to involve the peritoneum in inflammation, are also more fatal than those occurring in the perineum. The number of recoveries recorded after severe wounds of the bladder through the perineum, is truly wonderful.

On the subject of laceration of the bladder we have nothing to notice further than a careful account of the accident. In vesico-vaginal fistula our author adopts Sims' operation, and speaks of it and Dr. Sims in terms of the warmest commendation. Every step of this surgical procedure is carefully described, and illustrated by wood-cuts.

Inflammation, fibrinous exudation, ulceration, gangrene, and catarrh of the bladder, are next considered. In the treatment of this latter affection, Dr. G. speaks of copaiba as the most reliable remedy, but attributes occasional good effects to the terebinthinates, uva ursi, buchu, muriated tr. ferri, etc. The *pareira brava,* so great a favorite with English writers, has not met his anticipations. Of the injection of nitrate of silver he makes only casual mention. Within our own observation this means has been attended with a very marked success in the treatment of catarrhal and chronic cystitis, and it is so safe and easily applicable as to recommend it to early trial.

Under the head of "bar-like ridge of the neck of the bladder," we have a description of a thickening of the sub-mucous cellular tissue, stretching across from the openings of the ureters, (that is across the posterior margin of the trigone vesical,) usually attended with an enlargement of the third lobe of the prostate, or the uvula vesicæ. It is extremely difficult to diagnosticate, but being attended with catarrhal symptoms it does not require an essentially different treatment.

On the subject of stone in the bladder, and of prostatic enlargement, our author is very full. It is impossible for us, in a paper of this sort, to follow him through in an analytical way, but our readers will find that he has treated the subject in a very able manner, so much so as to render it the best treatise in the language.

An appendix is given containing a vast amount of information on the relative prevalence of stone in different localities, and the different chemical characters which obtain. We are rejoiced that Prof. Gross has given us this second edition in its present elegant form, and commend it unreservedly to our readers.

For sale by L. DANFORTH, 230 Main street.

